# EGF Receptor Exposed to Oxidative Stress Acquires Abnormal Phosphorylation and Aberrant Activated Conformation That Impairs Canonical Dimerization

**DOI:** 10.1371/journal.pone.0023240

**Published:** 2011-08-10

**Authors:** Simone Filosto, Elaine M. Khan, Emiliana Tognon, Cathleen Becker, Majid Ashfaq, Tommer Ravid, Tzipora Goldkorn

**Affiliations:** Center for Comparative Respiratory Biology and Medicine, Genome and Biomedical Sciences Facility, University of California School of Medicine, Davis, California, United States of America; Cornell University, United States of America

## Abstract

Crystallographic studies have offered understanding of how receptor tyrosine kinases from the ErbB family are regulated by their growth factor ligands. A conformational change of the EGFR (ErbB1) was shown to occur upon ligand binding, where a solely ligand-mediated mode of dimerization/activation was documented. However, this dogma of dimerization/activation was revolutionized by the discovery of constitutively active ligand-independent EGFR mutants. In addition, other ligand-independent activation mechanisms may occur. We have shown that oxidative stress (ox-stress), induced by hydrogen peroxide or cigarette smoke, activates EGFR differently than its ligand, EGF, thereby inducing aberrant phosphorylation and impaired trafficking and degradation of EGFR. Here we demonstrate that ox-stress activation of EGFR is ligand-independent, does not induce “classical” receptor dimerization and is not inhibited by the tyrosine kinase inhibitor AG1478. Thus, an unprecedented, apparently activated, state is found for EGFR under ox-stress. Furthermore, this activation mechanism is temperature-dependent, suggesting the simultaneous involvement of membrane structure. We propose that ceramide increase under ox-stress disrupts cholesterol-enriched rafts leading to EGFR re-localization into the rigid, ceramide-enriched rafts. This increase in ceramide also supports EGFR aberrant trafficking to a peri-nuclear region. Therefore, the EGFR unprecedented and activated conformation could be sustained by simultaneous alterations in membrane structure under ox-stress.

## Introduction

The epidermal growth factor receptor (EGFR, ErbB1) is a member of the ErbB family of receptor tyrosine kinases, which also includes ErbB2, ErbB3, and ErbB4. While these receptors have a critical role in normal cellular processes such as cell division, differentiation, and migration, their over-expression or dysregulation have been linked to a variety of human cancers, including breast, head and neck, lung, and ovarian [1], [2], [3]. As such, the activation of these receptors, particularly the EGFR, has been a subject of intense study.

A paradigm of EGFR activation has been established wherein ligand binding induces receptor dimerization, leading to the activation of its intrinsic tyrosine kinase activity, auto-phosphorylation, and subsequent phosphorylation of downstream signaling molecules [4], [5], [6], [7]. Recent advancements in crystallographic studies have demonstrated that EGFR dimerization occurs upon the binding of one EGF molecule to one EGFR, which releases the extracellular portion of the receptor from its “tethered” conformation. This exposes the otherwise buried “dimerization arm”, which can then interact with its dimerization partner in an entirely receptor-mediated back-to-back orientation [8], [9], [10], [11].

However, the dogma of EGFR dimerization/activation upon ligand binding has been challenged by the discovery of the L858R and other somatic mutations of EGFR that affect receptor conformation and sensitivity to tyrosine kinase inhibitors (TKIs), supporting the idea that EGFR can be activated without its ligand and without ligand-supported dimerization [12], [13], [14], [15], [16], [17].

Indeed, besides EGFR mutations, a few ligand-independent activations of EGFR have been described, such as via cigarette smoke [18], cell to cell interaction [19] or upon cholesterol-enriched lipid rafts disruption [20]. The physiological relevance of such poorly understood mechanisms has been recently brought to attention again by the work of Bublil et al [21], who demonstrated that EGFR exists in a “quasi-dimer” state that is stabilized by both extracellular and intracellular receptor-receptor interactions, whose formation does not require extracellular ligand. Also, Chung et al [22] demonstrated that unligated EGFR changes constantly between monomer and dimer states and pre-created dimers are ready for ligand binding and signaling. Consistently, ligand-independent dimers of EGFR were proven early on to be a step separable from ligand-induced downstream signaling [23].

Our previous studies demonstrated that CS-induced H_2_O_2_ generation or direct exposure to H_2_O_2_ cause phosphorylation of EGFR with a pattern of phosphorylation sites that differs from the one induced by EGF binding. More specifically, we reported that under H_2_O_2_-induced ox-stress EGFR is aberrantly phosphorylated, particularly at tyrosine (Tyr) Y845 and Y1045, which are hyper-phosphorylated and un-phosphorylated, respectively (in comparison to the EGF-stimulated receptor). This ultimately resulted in an active EGFR with impaired trafficking and degradation due to lack of ubiquitination and subsequent strong Src-dependent interaction with phosphorylated caveolin-1 and recruitment into caveolae and not clathrin coated pits. [18], [24], [25], [26].

Additional data suggested a correlation between tumor progression and altered cell redox status, but the underlying mechanisms were far from being understood [27].

Here, we present a novel model of ox-stress-induced EGFR activation which is ligand-independent and does not involve canonical dimerization. It appears to involve a conformational change in the intracellular kinase domain that is also dependent on temperature and membrane cholesterol/ceramide ratio and thus might be affected by changes in membrane structure/fluidity. We previously reported that under H_2_O_2_- or cigarette smoke-induced ox-stress membrane ceramide levels are increased [28], [29], [30], [31], which is known to alter membrane fluidity through cholesterol displacement [32]. Consistently, ceramide generation under ox-stress may displace cholesterol in membrane rafts and thus support changes in the EGFR conformation, whereas cholesterol uptake in the plasma membrane could inhibit such ox-stress-induced activation of EGFR.

In addition, we show herein that upon exposure to H_2_O_2_ active EGFR and active c-Src co-localize with elevated ceramide. Moreover, under such H_2_O_2_-induced ox-stress c-Src is physically bound to EGFR; this is not found under EGF treatment.

## Results

### H_2_O_2_ activates EGFR in a ligand-independent manner

**Figure 1:**
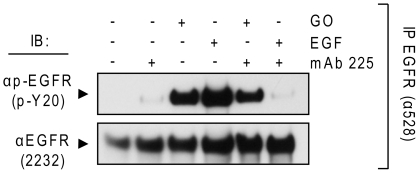
H_2_O_2_ activation of EGFR is ligand-independent. Serum-starved A549 cells were left intact or incubated with 40 nM monoclonal antibody 225 (mAb 225) for 1 hr on ice. Cells were then exposed for 15 min. at 37°C to 100 ng/ml EGF or 1 U/ml GO in the presence or absence of the mAb 225, as indicated. Immuno-precipitation (IP) of EGFR from cell lysates was performed using the mAb 528 and immuno-blotting (IB) for total (EGFR) and Tyr-phosphorylated EGFR (p-EGFR) was carried out as described in “Material and Methods”.

A549 cells were serum-starved overnight and, where indicated (Fig. 1), incubated with 40 nM monoclonal antibody (mAb) 225 for 60 min. on ice to block the EGFR ligand binding site. Cells were then exposed to either 100 ng/ml EGF for 15 min. or 1 unit (U)/ml glucose oxidase (GO; to generate H_2_O_2_) for 30 min. in the absence or presence of the mAb. EGFR was immuno-precipitated (IPed) from cell lysates and immuno-blotted (IBed) for assessing the total and tyrosine (Tyr)-phosphorylated (p-) EGFR levels. Phosphorylation of the EGFR by EGF was blocked by the mAb whereas phosphorylation by GO could not be inhibited (Fig. 1). This indicates that H_2_O_2_-induced EGFR phosphorylation is ligand-independent.

### H_2_O_2_ induces EGFR auto- and trans-phosphorylation

Thus far, it appears that H_2_O_2_ induces phosphorylation of the EGFR via a mechanism that is different from that induced by its ligand, EGF. One possibility is that H_2_O_2_ trans-activates EGFR by a non-receptor tyrosine kinase such as c-Src, which has been shown to be activated by ox-stress and to phosphorylate EGFR at Y845 [18], [24]. We, therefore, wondered whether EGFR auto-phosphorylation sites were also dependent on c-Src activation by H_2_O_2_.

**Figure 2 pone-0023240-g002:**
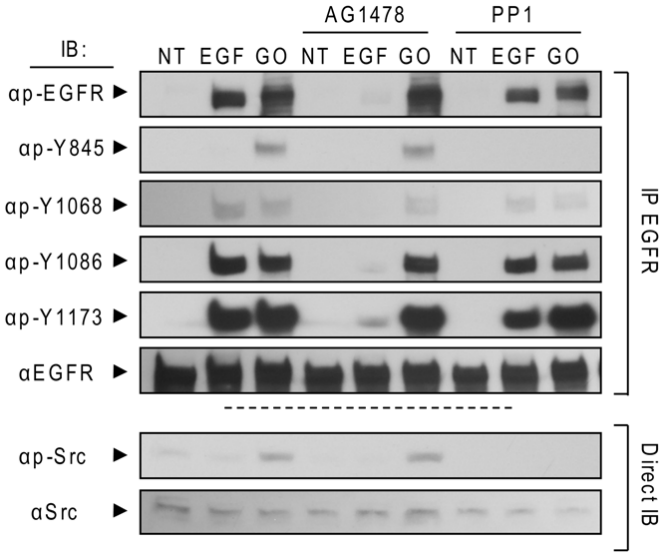
H_2_O_2_-induced EGFR phosphorylation is not inhibited by TKI AG1478 and is inhibited only at tyrosine 845 by Src family kinase inhibitor PP1. Serum-starved A549 cells were incubated (or not) with 1 µM AG1478 or 5 µM PP1 for 30 min. Then, the cells were treated for 30 min. with 100 ng/ml EGF or 1 U/ml GO, as indicated. EGFR was IPed from cell lysates, resolved by SDS-PAGE and IBed for total receptor, total tyrosine phosphorylation (p-EGFR) and specific Tyr-residue phosphorylation level (Y845, Y1068, Y1086, and Y1173). Protein aliquots of the cell lysates were also directly IBed for total and Y416 phosphorylated (active) c-Src (p-Src).

To answer this question we examined the effect of PP1, a specific inhibitor of the Src kinase family, on H_2_O_2_-induced phosphorylation of EGFR. Pre-treatment of A549 cells with 5 µM PP1 eliminated H_2_O_2_-induced trans-phosphorylation of EGFR at Y845 while auto-phosphorylation at Y1068, Y1086, and Y1173 remained unchanged (Fig. 2, last 3 columns vs first 3). These results indicate that H_2_O_2_-induced auto-phosphorylation is not dependent on Src-family kinases, which are only responsible for the trans-phosphorylation on Y845 (Fig. 2).

### H_2_O_2_-induced EGFR auto- and trans-phosphorylation are not inhibited by AG1478 in intact cells, but are inhibited in membrane fractions

The tyrosine kinase inhibitor (TKI) AG1478 can suppress EGFR auto-phosphorylation under EGF stimulation by reversibly blocking the ATP binding site of the EGFR kinase domain. Thus, we decided to test the efficacy of this TKI on H_2_O_2_-induced phosphorylation of EGFR.

Serum-starved A549 cells were incubated (or not) with 1 µM AG1478 for 30 min. and then treated (or not) with 100 ng/ml EGF or 1 U/ml GO for additional 30 min. Cells were lysed, EGFR was IPed and IBed for total receptor, total Tyr-phosphorylation and site specific Tyr-phosphorylation, as indicated in figure 2. EGF-stimulated cells showed a marked increase in phosphorylation compared to un-stimulated cells at Y1068, Y1086 and Y1173, known EGFR auto-phosphorylation sites, while trans-phosphorylation of Y845 was comparable to controls (Fig. 2). In contrast, treatments with 1 U/ml GO for 30 min. resulted in marked phosphorylation of Y845, Y1173, Y1068 and Y1086 compared to un-treated cells. Identical results were obtained when a concentration of 10 µM AG1478 was used (not shown).

Surprisingly, while pre-incubation with AG1478 was able to quench EGFR auto-phosphorylation upon EGF stimulation, it was not effective in the GO treated cells (Fig. 2, fifth vs sixth column). Since we previously showed that the kinase activity of EGFR is necessary for auto-phosphorylation of the receptor under ox-stress [26], this evidence suggested a conformational change in the kinase domain of EGFR under ox-stress, which prevents the inhibition of AG1478.

**Figure 3 pone-0023240-g003:**
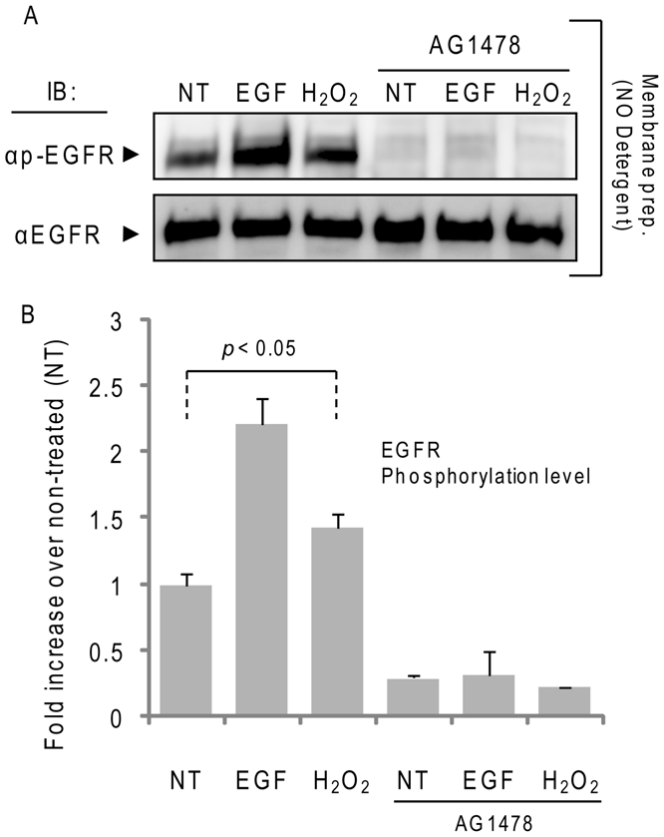
H_2_O_2_-induced EGFR phosphorylation is inhibited by TKI AG1478 in crude membrane fractions. A549 crude membrane fractions were prepared as indicated in “Material and Methods”. Membrane fractions were incubated with 1 µM AG1478 for 30 min. at 4°C and then incubated for 30 min. with 100 ng/ml EGF or 300 µM H_2_O_2_ at 37°C. **A**. Membrane proteins were separated by SDS-PAGE and IBed for total and Tyr-phosphorylated EGFR, as indicated. **B**. The histogram represents the levels of Tyr-phosphorylation of EGFR under different conditions, reported as fold-increase/decrease of the non treated (NT) sample; St-Deviations are indicated.

We further investigated the ability of the TKI AG1478 to suppress EGFR phosphorylation under ox-stress using crude membrane fractions of A549 cells, prepared as described in “Material and Methods”. Membrane fractions of equal protein content (10 µg each) were incubated (or not) with 1 µM AG1478 for 30 min. and then treated (or not) for additional 30 min. at 37°C with 100 ng/ml EGF or 300 µM H_2_O_2_. Samples were then separated by SDS-PAGE and IBed for total and Tyr-phosphorylated receptor (Fig. 3). AG1478 was able to completely inhibit both EGF- and H_2_O_2_-dependent EGFR phosphorylation (Fig. 3). In addition, AG1478 could inhibit both the EGF- and the H_2_O_2_-induced EGFR phosphorylation when crude broken cells were used instead of membrane preparations for the above *in vitro* phosphorylation assay (not shown).

The ability of AG1478 to inhibit H_2_O_2_-induced EGFR kinase activity in crude membrane fractions or in broken cells, but not in intact cells (Fig. 3 vs Fig. 2) further suggests that activation of EGFR by H_2_O_2_ involves a change in EGFR conformation that is different from the sequence of events following ligand binding and also implicates that the membrane integrity could be mediating or involved in stabilizing such a newly acquired conformation.

### EGFR phosphorylation by ox-stress is not accompanied by canonical receptor dimerization

**Figure 4:**
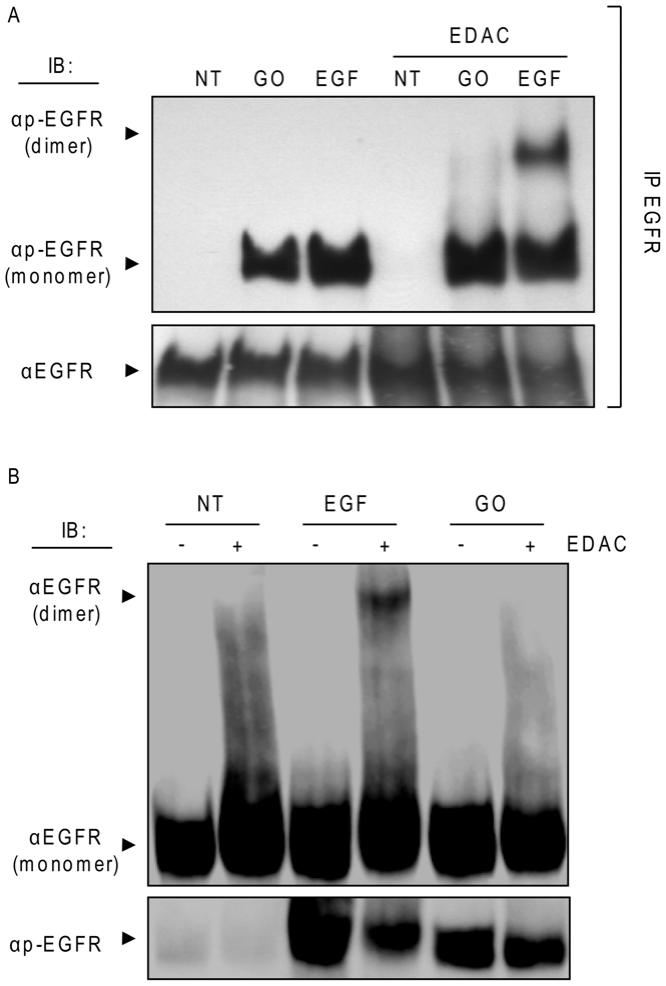
H_2_O_2_ activation of EGFR does not induce receptor dimerization. **A.** Serum-starved A549 cells were exposed (or not) to 100 ng/ml EGF or 1 U/ml GO for 15 min. Then, the cross linking reagent EDAC (1 mM) was added and incubation was continued for an additional 15 min. EGFR was IPed from cell lysates, resolved by SDS-PAGE and IBed for total and Tyr-phosphorylated EGFR as indicated; presence of EGFR dimers is indicated. **B.** Serum-starved NIH-3T3 cells over-expressing wild type EGFR were treated as in A; 50 µg of total cell lysates were IBed for total and Tyr-phosphorylated EGFR as indicated.

To examine whether ox-stress can induce EGFR dimerization, serum-starved A549 cells were left untreated or were exposed to 100 ng/ml EGF or 1 U/ml GO for 15 min. Then, the cross-linking agent, EDAC (1 mM), was added (or not) for an additional 15 min. EGFR was IPed from protein extracts, separated by SDS-PAGE and IBed for total and Tyr-phosphorylated EGFR (Fig. 4A). EGF treatment resulted in the formation of phosphorylated EGFR dimers, as seen in figure 4A at about 340 kDa. In contrast, ox-stress did not induce formation of EGFR dimers, both in the absence and presence of the cross-linking EDAC (Fig. 4A). In addition, NIH-3T3 cells over-expressing wild type EGFR were treated as above and total protein lysates were IBed for total and Tyr-phosphorylated EGFR as indicated in figure 4B. Again, EGF stimulation generated an EGFR dimer at ∼340 kDa, but ox-stress exposure (GO) did not (Fig. 4B), further supporting the idea that the ox-stress-activated EGFR acquires a conformation that differs from that of the EGF-stimulated one.

### c-Src binds activated-EGFR under ox-stress

Since we could not detect EGFR dimers under ox-stress using a plasma membrane impermeable zero spacer length cross-linking agent (EDAC) (Fig. 4), we wondered whether there was a conformational change within the intracellular domain of the EGFR that allowed its phosphorylation.

Of note is that wild type (WT) EGFR does not bind c-Src. However, it has been previously shown that a conformational change in the kinase domain of the EGFR due to somatic mutation (L858R) can result in a constitutive interaction with c-Src [33], [34]. Therefore, we investigated whether WT- EGFR could bind c-Src under ox-stress.

**Figure 5:**
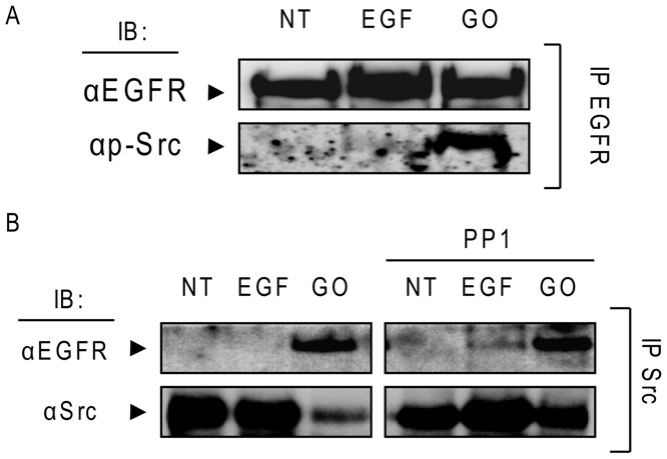
EGFR directly interacts with c-Src under H_2_O_2_ and such interaction is not abolished by Src family kinase inhibitor PP1. A549 cells were incubated (or not) with 5 µM PP1 for 45 min. and then treated (or not) for 15 min. with 100 ng/ml EGF or 30 min. 1 U/ml GO. **A**. EGFR was IPed from total cell lysates with the mAb 528 and IBed for Y416 phosphorylated c-Src (p-Src) and for total EGFR, as indicated. **B**. c-Src was IPed from total cell lysates and IBed for total c-Src and EGFR, as indicated.

Serum-starved A549 cells were treated (or not) with 100 ng/ml EGF for 15 min or 1 U/ml GO for 30 min. Then, EGFR was IPed, separated by SDS-PAGE and IBed for total EGFR and active (p-Y416) c-Src (Fig. 5A). At the same time an IP was carried out from the same samples using an Ab specific for total c-Src and respective IBs were performed against total EGFR and total c-Src (Fig. 5B). The results shown in figure 5 demonstrate that EGFR is bound by c-Src (active c-Src) under ox-stress (Fig. 5A and B). Moreover, we investigated whether such binding was dependent upon activation of c-Src by ox-stress. Therefore, A549 cells were pre-incubated, or not, with 5 µM PP1 (Src inhibitor) for 45 min. prior to stimulation with 100 ng/ml EGF (15 min) or 1 U/ml GO (30 min.) (Fig. 5B). PP1 inhibition of c-Src activation did not prevent the binding of c-Src to EGFR, and also did not lead to the conventional EGFR dimerization under ox-stress (data not shown). This demonstrates that activation of c-Src is not required for its interaction with EGFR under ox-stress and further supports the notion that an intrinsic conformational change in the EGFR exposed to ox-stress enables its binding to c-Src.

Of note is that c-Src is highly activated under ox-stress and thus may also be undergoing degradation. This is presently being investigated in our laboratory.

### Oxidative stress induces a novel active conformation of EGFR kinase domain

In order to support our hypothesis that under ox-stress EGFR acquires a novel active conformation, we tested the binding of a new EGFR monoclonal antibody, α4-2 mAb. This Ab is susceptible to the conformational changes of the activated EGFR kinase domain in non-denaturing conditions [35]. It recognizes amino acids 956–998, epitopes of EGFR that are exposed through the conformational change induced by EGF binding to the receptor. Of note is that same epitopes are also exposed in the constitutively active L858R EGFR mutant, and, therefore, such a mutant also binds efficiently the α4-2 mAb [35].

**Figure 6:**
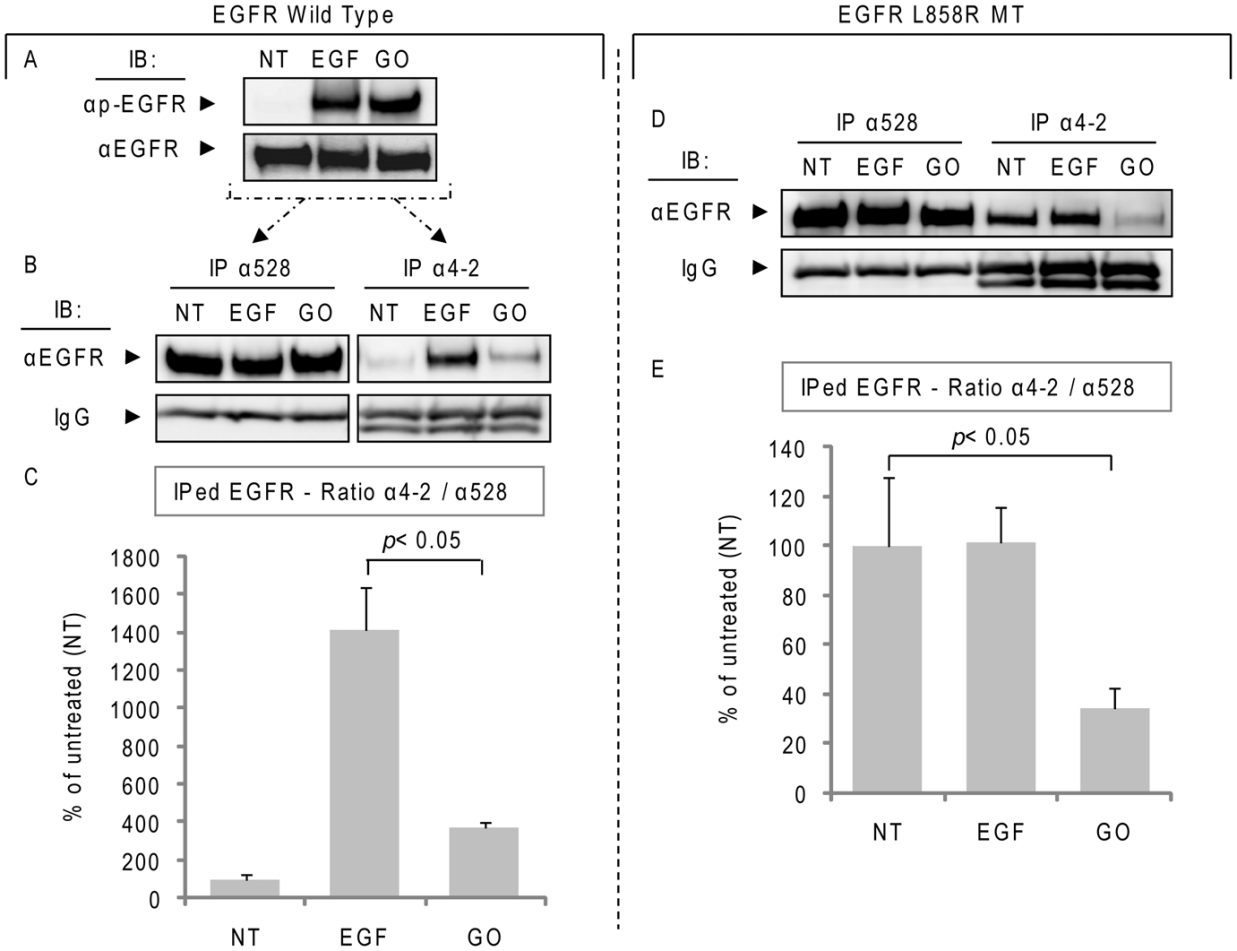
Oxidative stress induces a novel active conformation of EGFR. **A.** A549 cells were treated (or not) with 100 ng/ml EGF for 15 min. or with 1 U/ml GO for 30 min; Tyr-phosphorylation (p-Y20) and total EGFR levels were assessed by IB, as indicated. **B.** EGFR was IPed from 300 µg of the total cell lysates using either α528 or α4-2 Ab and then IBed with αEGFR Ab (2232). Samples IBs and the IgG heavy chains (stained with αmouse Ab) of the Abs used in the IPs are shown. The histogram in **C** represents the averaged ratio between total EGFR (IPed with α528 Ab) and the EGFR IPed with the α4-2 Ab (which has affinity for the “classical” EGF-induced active receptor conformation) of three independent experiments, quantified by densitometry of the bands; St-Dev are indicated. **D.** NIH-3T3 cells stably over-expressing L858R EGFR MT were treated as in A and EGFR was IPed as in B with either α528 or α4-2 Ab. Sample IBs of the IPs are shown for both EGFR (α2232) and IgG (αmouse Ab), as indicated. The graphic in **E.** represents the averaged ratio between total EGFR (IPed with α528 Ab) and EGFR IPed with the α4-2 Ab, quantified by densitometry of the bands (as in C); St-Dev are indicated.

As shown in figure 6, A549 cells were treated with EGF or GO (Fig. 6A), as before and EGFR was IPed from 300 µg of total cell lysates using 3 µg of either α4-2 Ab or α528 Ab and then IBed with αEGFR (2232) Ab to estimate the total EGFR that was IPed (Fig. 6B). Even though the activation level of EGFR was comparable under EGF or GO treatment, as assessed by the tyrosine phosphorylation level (αp-Y20)(Fig. 6A), the α4-2 Ab pulled down the EGF-stimulated EGFR with an affinity ∼3.5 folds higher than that observed under ox-stress (GO) activation (Fig. 6B–C). Control IgG heavy chains of the Abs used in the IPs are also shown (Fig. 6B). Furthermore, we investigated the ability of the α4-2 Ab to bind to the L858R mutant EGFR. For this purpose, we employed NIH-3T3 cells stably over-expressing the mutant [34]. These cells were exposed to EGF or GO and EGFR was IPed with either α4-2 or α528 Ab and IBed with αEGFR (2232) Ab, as described above. Figure 6D confirms that the α4-2 Ab binds efficiently and constitutively to the L858R EGFR MT, as previously reported [35]. However, when cells expressing that mutant were exposed to ox-stress (GO) the affinity of the L858R EGFR for the α4-2 Ab was reduced ∼70% in comparison to the untreated or EGF-treated cells (Fig. 6D–E). Therefore, the use of this novel Ab, directly demonstrates that the active conformation of EGFR under ox-stress differs from that of the EGFR stimulated by EGF. Moreover, the conformation of the EGFR mutant L858R under ox-stress is also being altered.

### H_2_O_2_ activation of the EGFR is temperature- dependent

We investigated the activation of EGFR by ox-stress (and by EGF) at different temperatures.

**Figure 7:**
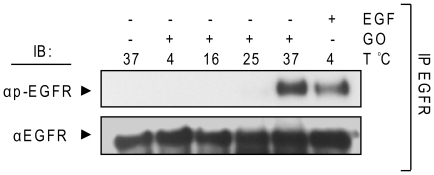
H_2_O_2_ activation of EGFR is temperature-dependent. Serum-starved A549 cells were left intact or incubated for 30 min. with 1 U/ml GO or 15 min. 100 ng/ml EGF at the indicated temperatures (T). EGFR was IPed from cell lysates and IBed for total and Tyr-phosphorylated EGFR, as indicated.

A549 cells were treated with 1 U/ml GO at 4, 16, 25, and 37°C or with 100 ng/ml EGF at 4°C. EGFR was IPed from protein extracts and IBed for total and Tyr-phosphorylated EGFR (Fig. 7). The data show that EGFR is activated by ox-stress only at 37°C whereas EGF activates EGFR even at 4°C (Fig. 7). Since H_2_O_2_ is generated by GO in a separate pre-incubation of the treatment media at 37°C (see methods), it is not likely that the lower treatment temperatures are interfering with H_2_O_2_ generation. Again, these results indicate that EGFR activation by ox-stress differs from its activation by the ligand, EGF, and could be dependent on membrane structure/fluidity.

### H_2_O_2_-induced activation of EGFR is inhibited by cholesterol up-take in the plasma membrane (PM)

Pike and Casey [36] suggested that localization of EGFR to lipid rafts confers a functional inhibition of the receptor and that depletion of cholesterol releases EGFR from such inhibition, leading to an increase in basal EGFR phosphorylation. Consistently, it was shown later that cholesterol depletion from PM results in increased EGFR phosphorylation [20]. Given the possible involvement of membrane structure in EGFR activation observed under ox-stress (Fig. 3 and 7), we investigated the potential involvement of cholesterol in such EGFR activation.

**Figure 8:**
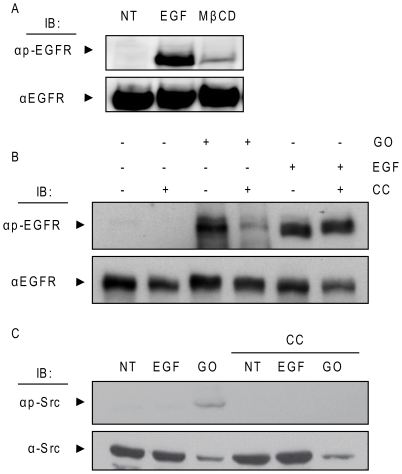
Cholesterol levels modulate EGFR activation by H_2_O_2_. **A**. Serum-starved A549 cells were treated, or not, with 100 ng/ml EGF for 15 min. or 2% (w/v) MβCD for 1 h and cell lysates were IBed for total and Tyr-phosphorylated EGFR. **B** and **C**. Cells were treated with EGF as in **A** or with 1 U/ml GO for 30 min. in the absence or presence of 2 mM MβCD-cholesterol complex (CC), prepared as described in “Material and Methods”. Cell lysates were separated by SDS-PAGE and IBed for total and Tyr-phosphorylated EGFR (**B**) or total and Y416 phosphorylated Src (**C**).

Others have shown before that disruption of lipid rafts by methyl-beta cyclodextrin (MβCD) treatment leads to EGFR activation and consequential distal signaling events [20]. Therefore, we first confirmed (Fig. 8A) that, indeed, cholesterol depletion from PM of A549 cells by MβCD treatment does activate EGFR, as was shown before [20]. Subsequently, A549 cells were treated with EGF or GO in the presence of 2 mM MβCD-cholesterol complex (CC) (prepared as described in “Material and Methods”), known to cause a substantial uptake of cholesterol in the PM [37]. The results show that cholesterol uptake (CC) significantly reduced the EGFR activation/phosphorylation under ox-stress exposure (GO) (Fig. 8B). At the same time, EGFR activation by EGF was just slightly changed by the addition of the MβCD-cholesterol complex (Fig. 8B).

**Figure 9:**
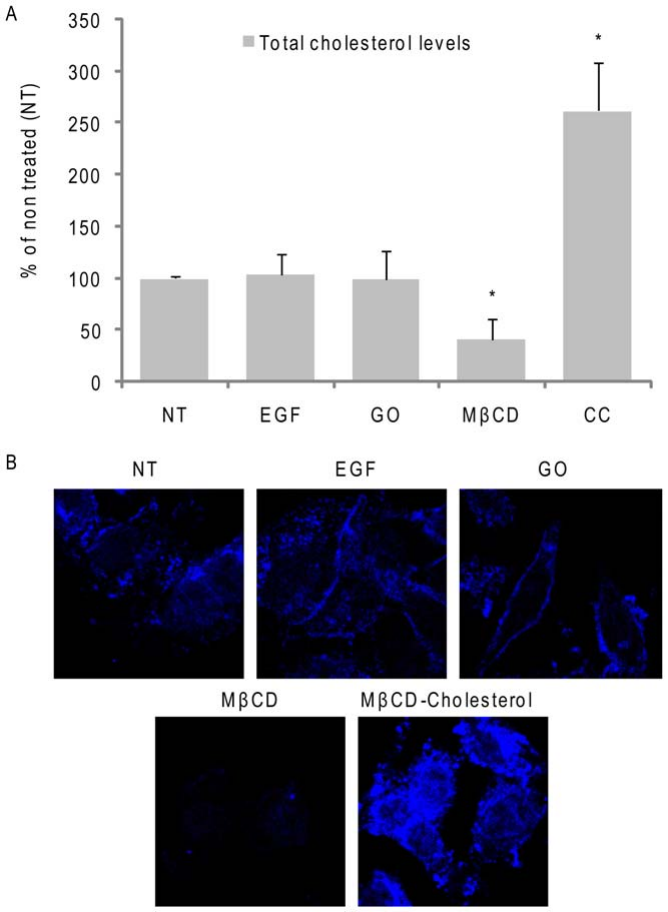
H_2_O_2_-induced ox-stress does not deplete cellular cholesterol. Serum-starved A549 cells were treated, or not, with 100 ng/ml EGF for 15 min. or 1 U/ml GO for 30 min. or 2% (w/v) MβCD for 1 h or 2 mM MβCD-cholesterol complexes (CC) for 30 min. **A**. Total cell cholesterol levels were measured after lipid extractions and normalized per protein unit, as described in “Material and Methods”; the values in the histogram are reported as % over non treated (NT) cells; St-Devs are indicated and “*” means p<0.05 in respect to control (NT); n = 3. **B**. Cells were fixed with paraformaldehyde (see “Material and Methods”) and stained for cholesterol using the sterol-binding probe filipin (50 µg/ml for 30 min.).

Interestingly, the uptake of cholesterol (CC) also inhibited the activation of c-Src under ox-stress (Fig. 8C), supporting the idea that not only EGFR activation under ox-stress could be regulated by membrane structure, but that also c-Src activity could be affected by such changes in the membrane. Finally, we measured the total cholesterol levels after the various treatments in cell lipid extracts (Fig. 9A), and also, by fluorescence microscopy (using the sterol-binding fluorescent dye filipin) in whole cells (Fig. 9B). As shown in figure 9, we confirmed that cholesterol was depleted from PM by MβCD treatment and that MβCD-cholesterol complexes substantially increased the amount of cholesterol in the PM. However, total cholesterol levels did not appear to be affected by GO treatment (nor by EGF).

### H_2_O_2_ elevates ceramide cellular levels, leading to both c-Src and EGFR co-localization with ceramide

Elevated ceramide levels have been shown to alter cell membrane structure and fluidity through displacement of cholesterol [32], [38], [39], and thus ceramide may act by physically changing the structure and properties of membrane lipid rafts.

Since we have being studying the mechanisms of ceramide generation under exposure to ox-stress [28], [29], [30], [40], [41], [42], we wondered whether ceramide levels were changed in our present studies and whether this alteration was coordinated within the same time frame of c-Src and EGFR activation under ox-stress exposure.

Serum-starved A549 cells were treated with 1 U/ml GO, as before, and then subjected to IF analyses by staining for ceramide, p-Y1173 EGFR or p-Y416 c-Src (as described in “Material and Methods”).

**Figure 10:**
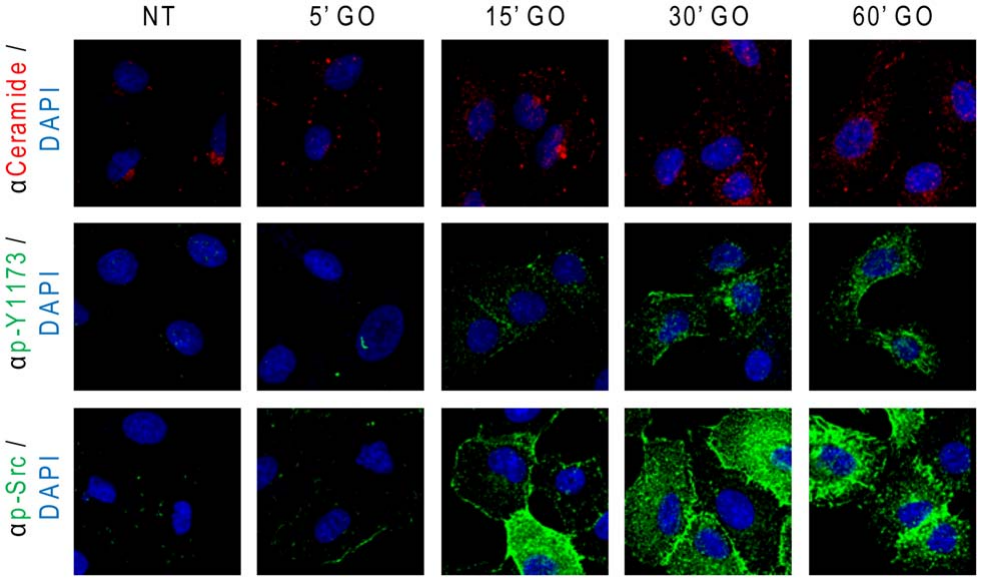
Ox-stress induces sustained increase of ceramide levels and activation of EGFR and c-Src. A549 cells were seeded on cover-glasses, serum starved for 1 h and treated (or not) with 1 U/ml GO as indicated. After treatments, ceramide, EGFR phosphorylated on Y1173 and Y416 phosphorylated c-Src were localized *in situ* by IF as indicated in “Material and Methods”; nuclei were stained by DAPI.

**Figure 11:**
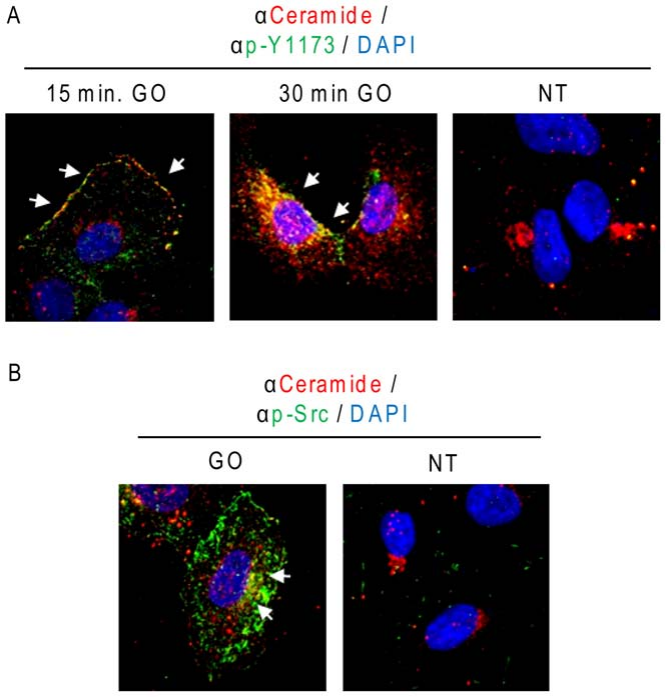
Under H_2_O_2_-induced ox-stress elevated ceramide co-localizes with active EGFR and c-Src. A549 cells were seeded on cover-glasses, serum starved for 1 h and treated (or not) with 1 U/ml GO as indicated. After treatments, ceramide (**A** and **B**), EGFR phosphorylated on Y1173 (**A**) and Y416 phosphorylated c-Src (**B**) were localized *in situ* by IF as indicated in “Material and Methods”; nuclei were stained by DAPI. White arrows indicate regions where p-Y1173 EGFR and ceramide (**A**) or p-Y416 c-Src (p-Src) and ceramide (**B**) co-localized under ox-stress (GO). Z-stack sections of cells have been discriminated by confocal microscopy: the panels show the merge of all of the Z-stack sections.

As shown in figure 10, increasing time points of exposure to GO elevated not only ceramide levels, but also the activation of EGFR (p-Y1173) and c-Src (p-Src). However, while under the addition of EGF a rapid activation of EGFR was followed by its rapid internalization via clathrin coated pits and lysosomal degradation [24], the activation of EGFR under ox-stress was sustained and increased over time (Fig. 10). Subsequently, we aimed to elucidate the kinetics of co-localization between the generated ceramide and the ox-stress activated EGFR. Using confocal microscopy, we demonstrated (Fig. 11) that after 15 min. of exposure to ox-stress (GO), active EGFR (p-Y1173) and the elevated ceramide levels were localized to the plasma membrane, where the two signals merged (white arrows in Fig. 11A). However, after 30 min. of GO exposure, the elevated ceramide and the activated EGFR were observed in a “peri-nuclear” cell region (Fig. 11A). In addition, at that time point, the co-localization between active EGFR and ceramide was mainly observed at the peri-nuclear region (arrows in Fig. 11A). Furthermore, at that time point the GO-activated c-Src (p-Src) also co-localized with ceramide mainly in the peri-nuclear region (arrows in Fig. 11B). Since we have shown before [26] that under ox-stress activated EGFR traffics via caveolae to a peri-nuclear region, we suggest there is a role for ceramide generation in facilitating the recruitment of H_2_O_2_-activated EGFR into caveolae trafficking to peri-nuclear ceramide-enriched membrane domains as discussed below.

## Discussion

We have shown before that ox-stress exposure of lung epithelial cells induces aberrant EGFR phosphorylation, resulting in its lack of ubiquitination by c-Cbl and its impaired degradation. Subsequently the stabilized ox-stress activated EGFR remains plasma membrane-bound, while a portion of it is trafficked via caveolae to the peri-nucleus. In the present paper, we are starting to dissect the upstream molecular alteration in the structure/conformation of EGFR itself following exposure to ox-stress.

Data presented herein substantiate the findings that EGFR activation/phosphorylation under ox-stress is aberrant, thereby leading to a distorted activated conformation. Indeed, the supporting molecular data is as follows:

- EGFR binds c-Src, which does not happen when the receptor is activated by EGF.
- EGFR may dimerize, but because the activated conformation of the receptor is unique, and different than the canonical conformation obtained by EGF induction, the dimer cannot be cross-linked by a conventional cross-linker, such as EDAC.
- Acquiring a non-canonical activated conformation under ox-stress also prevents the inhibition by the tyrosine kinase inhibitor (TKI), AG1478. However, this TKI manages to fully inhibit EGFR phosphorylation in broken cells when the unique ox-stress acquired conformation is demolished.
- Finally, EGFR activated by ox-stress could not bind to a new Ab that recognizes only the EGF-induced active conformation of EGFR (or that of the EGFR activated by somatic mutations, such as the L858R [35]) because the epitopes recognized by this Ab are in the intracellular domain of EGFR, which are exposed only in these active conformations. EGFR activated under ox-stress, evidently attained a different conformation that did not expose these epitopes, and thus the conformation-specific Ab could not bind EGFR in its unique conformation under ox-stress.

Recent data from Chung et al [22], as well as the work of Bublil et al [21], demonstrated that EGFR does not need an extracellular ligand to form dimers. EGFR continuously changes from a monomer to a dimer state, where the interactions between the intracellular domains are as much of importance as that of the extracellular regions of the receptor in forming such dimers. This supports the significance of better understanding the mechanisms involved and the physiological relevance of processes leading to ligand-independent activation of EGFR.

In the present study we used A549 adenocarcinoma or NIH-3T3 cells stably over-expressing either the wild type or the L858R EGFR MT and several biochemical techniques to provide new insight into the mechanism of Tyr-phosphorylation/activation of EGFR under ox-stress.

Reports by others demonstrated the inactivation of protein tyrosine phosphatases (PTPs) by H_2_O_2_ and suggested that this was responsible for EGFR phosphorylation [43], [44], [45]. For example, data published by Xu et al indicated that H_2_O_2_ induced phosphorylation of EGFR on Y1068 while PTPs activity was reduced to ∼70% of control. However, we and others showed that H_2_O_2_ did not induce phosphorylation of a kinase-dead EGFR [26], [46], demonstrating that the kinase activity of the receptor is required for its activation by H_2_O_2_ and that a bulk inactivation of PTPs cannot explain the phenomenon.

Here we show that EGFR activation by H_2_O_2_ is a ligand-independent event that is not accompanied by “classical” receptor dimerization and is not inhibited by the TKI AG1478, indicating a novel unprecedented active conformation of EGFR that differs from that of the EGF-activated one (Fig. 1–4).

Another indication for a unique conformational change of EGFR under ox-stress was supported by the finding that EGFR was strongly associated with c-Src. Moreover, the interaction between EGFR and c-Src was not dependent on the activation of c-Src because it persisted even in the presence of the c-Src kinase inhibitor PP1 (Fig. 5).

Consistently, it was reported that c-Src stably interacts with ErbB2, but not with EGFR/ErbB1, because of differences in the kinase domains of the two receptors [47]. Additionally, studies with the L858R EGFR mutant (MT) also demonstrated that this MT could bind c-Src, whereas the wild-type (WT) EGFR could not (under physiological conditions). This MT has been crystallized and shown to possess a protein conformation that differs from that of the WT EGFR at the level of the kinase domain, carrying a constitutively open “activating-loop”. Interestingly, the L858R EGFR MT was subsequently shown to have a similar functional phenotype to that of the WT EGFR under ox-stress, previously described by our group [24], [25], [26], i.e. hyper-phosphorylation/activation, impaired trafficking, lack of ubiquitination and degradation and constitutive interaction with c-Src, without any ligand stimulation [15], [33], [34], [48]. This further supported the idea that H_2_O_2_ induces a conformational change in the intracellular kinase domain of EGFR. Accordingly, dimerization of the extracellular domain could not be captured by the EDAC cross linker neither for the wild type EGFR under ox-stress (Fig. 4), nor for the L858R MT (unpublished observations).

At the same time, even though EGFR under ox-stress appears to acquire a novel activated conformation, such a conformation seems to be different from that of the L858R EGFR MT, which is known to be sensitive to TKI [49].

Consistently, by employing a novel EGFR antibody (α4-2 mAb) that is susceptible to the conformational changes induced by EGF binding to the receptor [35] or by the somatic mutation L858R, we confirmed that H_2_O_2_ induces a unique active conformation of the receptor (Fig. 6), which is different from that of both EGF-induced EGFR and the L858 MT.

Hetero-dimerization of EGFR with other members of the ErbB family could possibly be different under ox-stress exposure. However, no high molecular dimers are observed, what so ever, in figure 4. At the same time, the cross linker EDAC used in our study had been shown by others [50] to be able to crosslink the hetero-dimer EGFR-ErbB2. Therefore, the likelihood of ErbB2 functioning as the activating kinase that phosphorylates EGFR under exposure to ox-stress is being excluded. Furthermore, we repeated all our studies in NIH-3T3 cells stably transfected with EGFR (not shown). It was previously reported by others that these cells “do not express” or “do not express significant amount of” ErbB family members [51], [52]. However, following ox-stress exposure, the same aberrant phenotypes of EGFR were obtained, indicating that EGFR aberrant phosphorylation, lack of dimerization, novel active conformation and the subsequent resistance to TKI AG1478 cannot be attributed to any irregular association of EGFR with any other member of the ErbB family under ox-stress exposure.

Since EGFR phosphorylation by H_2_O_2_ is shown to be temperature dependent, suggesting a requirement for membrane involvement, it was not surprising to find that the TKI AG1478 was ineffective in quenching EGFR phosphorylation by H_2_O_2_ in living cells, but capable of inhibiting it in a crude membrane fraction, where the membrane structure was destroyed. This supports our novel suggestion that the fluidity/structure of the membranes may be involved in either inducing or stabilizing the new H_2_O_2_-induced active conformation of EGFR. Therefore, we further investigated the participation of membrane constituents in EGFR activation by H_2_O_2_.

It was previously reported that disruption of cholesterol-enriched lipid rafts causes ligand-independent activation of EGFR [20]. One of the widespread explanations was that a population of EGFR is strongly associated with lipid rafts and their disruption by a cholesterol sequestering agent, such as MβCD, would cause the re-localization of EGFR in non-raft portions of the plasma membrane where it could be activated due to its release from raft-associated inhibiting factors [53]. However, recent studies suggested the existence of at least two kinds of raft populations, the cholesterol-enriched- and the ceramide-enriched-rafts. The ceramide-enriched rafts are typically generated through cholesterol displacement by ceramide and are “less fluid” [54]. Therefore, we are now proposing a new role for ceramide generation under ox-stress exposure in the process of EGFR aberrant activation and subsequent trafficking via caveolae to a peri-nuclear region, where it remains active (Fig. 10–11). Over the last ten years our group has been investigating the mechanism of ceramide generation under ox-stress in human airway epithelial (HAE) cells, showing that ceramide levels are increased not only in the membranes of HAE cells, but also in the lungs of mice exposed to H_2_O_2_ generated by cigarette smoke [28], [29], [31], [40], [41], [55]. At the same time others have shown that ceramide alters membrane fluidity and causes cholesterol displacement from lipid rafts. Finally, it was suggested that ceramide can induce the merging of lipid rafts in bigger signaling ceramide-enriched membrane platforms [32], [38], [39], [54], [56], [57].

Since we were not able to demonstrate cholesterol depletion induced by ox-stress (Fig. 8), we suggest that cellular ceramide generated (as a result of exposure to ox-stress) may just displace cholesterol from the rafts, thereby disrupting cholesterol-enriched rafts and leading to EGFR re-localization to the more rigid ceramide-enriched rafts.

We show here for the first time that ox-stress-activated EGFR, as well as activated c-Src, co-localize within such ceramide enriched regions. At early time points of ox-stress exposure (15 min.) active EGFR and elevated ceramide co-localize primarily in the plasma membrane of the cells. Later on (30 min), the co-localization is observed mainly in a peri-nuclear region of the cells. Interestingly, we have previously shown that ox-stress activated EGFR, unlike the EGF-stimulated receptor, is not internalized via clathrin-coated pits; while it is not degraded and remains active, it can then traffic via caveolae to the peri-nucleus because of strong association with phosphorylated caveolin-1 (Cav-1) under ox-stress [26]. Moreover, the ox-stress activated EGFR co-localized with the early endosomes marker EEA-1 [25], [26] and the recycling endosome marker rab-11 (unpublished observation). Taken together, the generation of ceramide, followed by cholesterol displacement may have a role in the aberrant trafficking of EGFR via caveolae to the peri-nuclear region under ox-stress exposure. Consistent with our hypothesis, it has been recently shown that ceramide generation increases the recruitment of Cav-1 into caveolae [58], [59], Given the well established patho-physiological function of EGFR and ceramide in carcinogenesis and severe lung injury, respectively [55], [60], [61], [62], [63], [64], [65], [66], [67], this evidence may have far reaching implications, which will drive further investigations in this direction.

**Figure 12:**
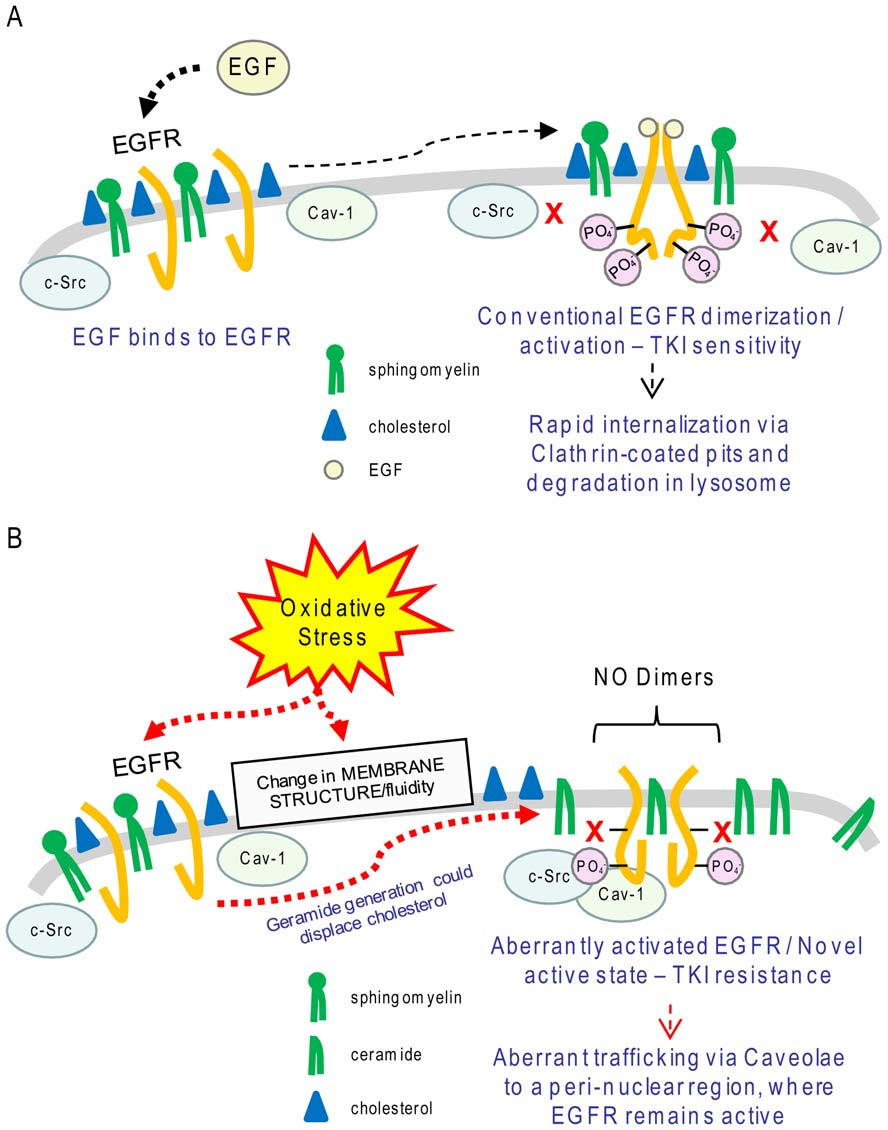
Proposed model of EGFR activation under ox-stress. **A.** Conventional activation/dimerization of EGFR upon stimulation by the ligand EGF. **B.** Ox-stress could cause a change in membrane structure/fluidity by increasing cellular ceramide levels, which could affect cholesterol distribution. This, together with possible direct effect of ox-stress on EGFR, induces, or stabilizes, a novel acquired active conformation of EGFR that is bound by active c-Src and caveolin-1 (Cav-1). Such aberrantly active EGFR does not dimerize “conventionally” and becomes resistant to TKI drug, while it traffics via caveolae to an unidentified peri-nuclear region, where it remains active. Please note: Cav-1 binding to EGFR under ox-stress was demonstrated by our group before [26].

In conclusion, data presented herein demonstrate that EGFR in cells exposed to ox-stress is not only aberrantly phosphorylated but it also acquires a novel conformation, which supports a ligand-independent mechanism of activation that does not require conventional receptor dimerization and is not inhibited by the TKI AG1478. These alterations in EGFR conformation are accompanied by c-Src binding to the receptor (Fig. 5). Whether the change in EGFR conformation under ox-stress occurs as a result of simultaneous alterations in the membrane structure, or happens independently and is only being stabilized by the simultaneous membrane changes requires additional studies (see model in Fig. 12).

## Materials and Methods

### Cell culture, treatments and reagents

A549 adenocarcinoma (ATCC) and NIH-3T3 cells have been employed in this study. A549 cells were cultured in F12K medium (GIBCO) supplemented with 10% FBS (GIBCO) and 1% pen/strep (GIBCO). NIH-3T3 cells were stably transfected with either the wild type (WT) EGFR or with the L858R EGFR mutant (these cells were kindly provided by Dr. H. Band, University of Nebraska Medical Center [34]) and were cultured in DMEM (GIBCO) medium supplemented with 10% FBS and 1% pen/strep. DMEM high glucose medium (GIBCO) was used for the treatments with no FBS supplementation. EGF was added directly into the treatment medium at a final concentration of 100 ng/ml. Glucose oxidase (GO, from Sigma) was used to generate H_2_O_2_, under conditions that were previously optimized [18], [24], [26]: briefly, DMEM medium was incubated for 15 min. at 37°C with 1 U/ml GO prior to being added on top of sub-confluent (∼70%) cells for an additional 15 min. For longer treatments the GO-medium was replaced every 15 min. 1-Ethyl-3-(3-dimethylaminopropyl) carbodiimide hydrochloride (EDAC) cross linker agent (Thermo Scientific) was dissolved in phosphate buffer saline (PBS) and then added to the treatment medium at a final concentration of 1 mM. Methyl-beta-cyclodextrin (MβCD, from Sigma) was dissolved directly in treatment medium at a final concentration of 2% (w/v), prior to putting such treatment medium on top of sub-confluent cells. PP1, a Src family inhibitor (Enzo Life Sciences), was dissolved in DMSO and added to the treatment medium at a final concentration of 5 µM. Tyrosine kinase inhibitor (TKI) AG1478 (Cell Signaling) was dissolved in DMSO and then added to the treatment medium at a final concentration of 1 µM. For MβCD-cholesterol complex (CC) preparation, 9% (w/v) MβCD was dissolved in PBS and heated while mixing at 80°C; cholesterol (Matreya) was slowly added to the heated solution until the MβCD was completely saturated by cholesterol (cholesterol is no longer solubilized); then the solution was filtered through 0.2 µm pores and added to the treatment medium with a final concentration of ∼2 mM MβCD-cholesterol complexes. PBS or DMSO was added at the appropriate concentration to the control-untreated cells where needed. Cells were collected by scraping in either PBS or directly in the lysis buffer: 1% NP-40 (Igepal, from Sigma), 50 mM Tris, 10% Glycerol, 0.02% NaN_3_, 150 mM NaCl, pH 7.4, containing a cocktail of phosphatase and protease inhibitors (Sigma) as well as 1 mM NaF and Na_3_VO_4_. Lysates were passed 5 times through a 30 gauge needle prior to centrifugation and further processing of the samples (either by immuno-precipitation or immuno-blotting).

### Sodium dodecyl sulfate polyacrylamide gel electrophoresis (SDS-PAGE)

5, 6, 8, 10 or 12% acrylamide gels were prepared following common procedures (not described) and run via a 2 Cell system (BioRad) for 1–4 h at 100 V at room temperature (RT).

### Immuno-precipitation (IP)

200–400 µg of total protein extracts were incubated overnight with 2–4 µg of antibodies (Abs): α528 (anti (α) EGFR Ab), αc-Src (Santa Cruz Biotech) or α4-2 (αactive-EGFR [35], kindly provided by Dr. K. Omi, Fujirebio Inc., Tokyo, Japan). 50 µl of 50% protein A-agarose bead complexes (Repligen) were added to the samples and incubated for 90 min. Four washes (by sequential centrifugation and re-suspension) with the NP-40-lysis buffer were done prior to re-suspending the IPs in 50 µl of 2× loading dye (for SDS-PAGE, see below).

### Immuno-blotting (IB)

20–100 µg of total protein extracts or the IP samples were loaded into each well of the SDS-PAGE in the presence of a sodium dodecyl sulfate (SDS)/dithiothreitol (DTT) reducing loading dye (common concentrations/conditions – samples heated for 5 min. at 95°C). After SDS-PAGE separation the proteins were transferred to a nitrocellulose membrane and “blocked” with 5% skim milk in tris buffered saline with 0.05% tween-20 (TBST) for 120 min. or overnight. Primary Abs were incubated in 5% milk-TBST for 2 h at RT. Secondary Abs, either goat αmouse- or goat αrabbit-horseradish peroxidase (HRP) conjugated (Jackson ImmunoResearch), were incubated for 90 min. at RT at 1∶10000 dilution in 5% milk-TBST. Bands were visualized by enhanced chemiluminescence (ECL, PIERCE). Extensive washes in TBST were done in between each step. Primary Abs used in this study for the IBs were: α2232 (αEGFR, Cell Signaling, 1∶1000), αc-Src (Santa Cruz Biotech, 1∶2000), α tyrosine Y416 phosphorylated (p-) c-Src (Cell Signaling, 1∶2000), αp-Y20 (Santa Cruz Biotech, 1∶3000), αp-Y1173 EGFR (Santa Cruz Biotech, 1∶1000), αp-Y1086, αp-Y1068 and αp-Y845 EGFR (Cell Signaling, 1∶1000).

### *In vitro* kinase assay

Cells were scraped in a detergent-free buffer (50 mM Tris-Base 0.02% NaN_3_ 1 mM EDTA in PBS) in the presence of protease and phosphatase inhibitors (Sigma), 1 mM NaF and Na_3_VO_4_, and then homogenized by several passages in a 30 gauge needle. Unbroken cells and nuclei were removed by a 5 min. centrifugation at 500×g at 4°C; then, the homogenates were spun down by centrifugation at 60,000×g (Beckman ultra-centrifuge) for 30 min. to isolate the insoluble/membrane (pellet) fraction from the soluble/cytosol (supernatant) fraction. Membrane preparations were resuspended in a reaction mixture of 1 mM MnCl_2_, 20 mM Hepes pH 7.4, 5 µg/ml BSA, 5 µM ATP, and incubated, or not, for 45 min. with 1 µM AG1478 at 4°C, prior to the addition (or not) of either 100 ng/ml EGF or 300 µM H_2_O_2_ (stock solution in water from Sigma). The reaction was carried out at 37°C for 20 min. and stopped by the addition of the SDS-PAGE loading dye. Alternatively, the cells were scraped directly in 1 mM MnCl_2_, 20 mM Hepes pH 7.4, 5 µg/ml BSA, 5 µM ATP and homogenized by several passages in a 30 gauge needle; unbroken cells and nuclei were removed by centrifugation for 5 min. at 500×g and the supernatant, containing the broken cells, was used directly for the in vitro kinase assay (without 60,000×g centrifugation step).

### Measurement of cholesterol levels

Cell cholesterol levels were measured by using a quantitative assay from Cell Technology, following manufacturer instructions. Briefly, after the treatments, A549 cells were washed, scraped in PBS and divided in two aliquots. One aliquot was used to determine total protein amount (normalization step) and to test the treatments. The other aliquot was used to extract total lipids and measure cholesterol levels. Lipids were extracted with chloroform: methanol (2∶ 1) then mixed with 1 M NaCl, prior to phase separation via centrifugation (5 min. at 1500×g). Lipids in the organic phase were collected, dried under nitrogen and resuspended in 0.1 M KPO_4_, 50 mM NaCl, 5 mM deoxycholic acid, 0.1% Triton-x 100. Aliquots of the resuspended lipids were measured for cholesterol content in the reaction mixture (Cell Technology protocol).

### Immuno-fluorescence (IF)

A549 cells were seeded on cover-glasses, treated (or not) with 1 U/ml GO for different exposure times and then processed for IF as previously described [18]. Primary Abs used in this study were: αp-Y416 c-Src (Cell Signaling, 1∶200); αp-Y1173 EGFR (Cell Signaling, 1∶50); αceramide*1(Alexis Biochem, 1∶20). Secondary Abs were Alexa Fluor 488 or 555, either goat αmouse or goat αrabbit 1∶200 dilution. Nuclei were stained with 4′,6-diamidino-2-phenylindole (DAPI). Cholesterol in fixed cells was stained by 30 min. incubation with 50 µg/ml filipin (Sigma). Cells were mounted on slides using “fluoromount-G” (Southernbiotech) and images were acquired by confocal microscopy using an Olympus FluoView FV1000 or a LSM 5 Pascal Zeiss system with 400× (air) or 630× (oil) magnification objectives. Observations were repeated at least three times independently by different scientists.
